# A comparison of heparinised and non-heparinised normal saline solutions for maintaining the patency of arterial pressure measurement cannulae after heart surgery

**DOI:** 10.1186/s13019-019-0860-8

**Published:** 2019-02-26

**Authors:** Jianqiu Xiong, Tuo Pan, Hua Jin, Xiaoli Xie, Yan Wang, Dongjin Wang

**Affiliations:** 1 Department of Cardio-Thoracic Surgery, Nanjing Drum Tower Hospital, the Affiliated Hospital of Nanjing University Medical School, Nanjing, 210008 Jiangsu China; 2 Department of Cardio-Thoracic Surgery, Drum Tower Hospital Clinical Medical College of Nanjing Medical University, Nanjing, 210008 Jiangsu China

**Keywords:** Normal saline, Heparin, Occlusion, Coagulation disorder

## Abstract

**Background:**

Heparinized solution (HS) use for the maintenance of arterial cannulas has been associated with coagulation disorders and has not been conclusively shown to confer additional benefits over normal saline (NS) alone. We tested the hypothesis that in adult patients admitted to the cardiac ICU (T0) after cardiac surgery, HS arterial cannulas might be independently associated with increased heparin-induced coagulation disorders and might not be superior to NS arterial cannulas in reducing arterial cannula occlusion.

**Methods:**

In this cohort study, 170 patients who received NS arterial cannulas during the period from T0 to ICU discharge were included in this study from June 1, 2017, to May 1, 2018 (NS group). There were 2930 patients who used HS (2.5 U/ml) arterial cannulas from January 1, 2015, to December 31, 2016 (heparin group). To address indicated biases, we derived a propensity score that predicted the functions of NS and HS in the patency of arterial cannulas.

**Results:**

There were 296 patients (148 in the NS group and 148 in the heparin group) with similar risk profiles in the propensity-score matched cohorts. In the propensity-matched patients, the duration of arterial cannulas (*P* = 0.4) and arterial cannula occlusion (*P* = 0.5) showed no differences between the NS and heparin groups. However, the heparin group had a significantly increased activated clotting time (*P* < 0.05), activated partial thromboplastin time (*P* = 0.01) and allogeneic red blood cell utilization (3.4% vs 10.8%, *P* < 0.05). Compared with the NS group, the heparin group had more drainage from chest tubes from T0 to T48 (10.6 ± 9.4 ml/kg vs 13.0 ± 7.22 ml/kg, P < 0.05) and had more allogeneic red blood cells transfused (0.1 ± 0.4 U vs 0.4 ± 1.1 U, P < 0.05).

**Conclusion:**

Based on the results of our study, the addition of heparin to normal saline for flushing arterial pressure monitoring cannulae did not reduce the incidence of catheter thrombosis and result in a very small but statically significant in increase in activated clotting time and activated partial thromboplastin time.

## Background

Cardiac surgery is increasingly performed for high-risk patients referred for operations [1]. Many attempts have been undertaken to reduce the mortality. Therefore, most patients who undergo cardiac surgery while admitted to a cardiac intensive care unit (CICU) routinely require arterial cannulas to enable accurate monitoring of systemic blood pressure and regular blood sampling [2]. However, the placement of arterial cannulas is not without associated complications. These complications, including cannula occlusion, vascular injury and bleeding, can occur early [3]. In particular, the replacement of arterial cannulas increased the risk of vascular injury and bleeding if the cannulas were frequently occluded.

Routine arterial cannula maintenance includes regular flushing with heparin solution (HS, heparin plus 0.9% sodium chloride) to prevent cannula occlusion [4]. Other cannula maintenance procedures include the use of infusions under positive pressure to prevent retrograde blood flow [4–6]. Heparin is a powerful drug in terms of its ability to prevent clots from forming in the cannula, but its use is not without risk of bleeding, allergic reactions and low platelet counts [7]. It has been reported that heparin treatment produced heparin-induced thrombocytopenia (HIT) in seriously ill patients [7]. Additionally, it is estimated that the occlusion rate is between 0 and 33% when using HS solution [8, 9]. Therefore, the risk of heparin exposure should also be emphasized.

Some studies, including a guideline [10], several trial [11–13], and a Cochrane review [4], have indicated that normal saline (NS) is safe and feasible for preventing cannula occlusion in adult patients. However, the Cochrane review reported that the available evidence was of poor quality to support the effects of adding heparin (1 to 2 U/mL) to a maintenance solution (pressurized to deliver 3 mL of flush solution per hour) of 0.9% normal saline in maintaining the patency and functionality of arterial cannulas [4]. Moreover, these studies did not conclusively show that HS conferred additional benefits over NS alone, especially if NS arterial cannulas decreased heparin-induced coagulation disorders (HICDs) in cardiac patients. We hypothesized that, in adult patients admitted to the cardiac ICU (T0) after cardiac surgery, NS arterial cannulas might be independently associated with decreased HICD, which might not be superior to NS arterial cannulas in maintaining the patency of arterial cannulas. Therefore, this study is aimed to compare the efficacy and safety of heparin and non-heparinised NS to maintain the patency of arterial pressure monitoring cannulae following heart surgery. In addition, to address any indicated biases, we derived a propensity score predicting the functions of NS and HS in postoperative outcomes.

## Methods

This study was approved by the ethical committee of Nanjing Drum Tower Hospital on February 28, 2017. From January 1, 2015, to May 1, 2018, 3100 patients aged > 18 years old and requiring NS and HS arterial cannulas were enrolled following surgery for the repair of heart disease in our Hospital. Patients with aortic diseases, acute mesenteric ischaemia, pregnancy, cancers, chronic obstructive pulmonary disease (COPD) and extracorporeal membrane oxygenation (ECMO) initiation before surgery, known allergies to heparin, platelet treatment, a history of HIT, coagulation disorder before using heparin and thrombocytopenia with platelets less than 100,000 mm3 were also excluded. After the study was approved by the ethical committee of our hospital, we reviewed hospital medical records, nursing records, the laboratory database, and the cardiac surgical database.

In our hospital, the replacement of heparinized arterial cannulas was routinely implemented before 2017. Therefore, based on the retrospective review of our institution’s database, 2930 patients who received heparinized arterial cannulas met the criteria from January 1, 2015, to December 31, 2016 (heparin group). To investigate whether NS arterial cannulas could improve outcomes, we selected 170 patients who underwent “simple” cardiac surgery and received postoperatively indwelling NS arterial cannulas since June 1, 2017 (NS group). These “simple” cardiac surgery cases included isolated coronary artery bypass grafts (CABGs), single-valve surgeries (SVS), and isolated CABG+ SVS.

### Materials and drugs

An arterial measurement transducer was acquired Edwards Lifesciences (PX260, Edwards Lifesciences, Dominican Republic). The types of arterial cannula were 20 G, 22G and 24 G (diameter: 1.1 mm, 0.9 mm and 0.7 mm). The arterial cannulas were made by Becton Dickinson Medical Devices Co. Ltd. (Suzhou No. 5 Baiyu Road, Suzhou Industrial Park, Jiangsu P.R. China). The transducers were calibrated, zeroed and levelled at the beginning of each working shift and also when the measurements demonstrated any problems. The radial artery was used for catheterization in all of the patients. To maintain homogeneity between the groups, radial arterial cannulas were replaced in these patients they had femoral or brachial arterial cannulas. The data collected by radial arterial cannulas were used for this study. Inflating the pressure infuser bag to a constant 300 mmHg has been routinely used to prevent retrograde blood flow. The catheterization technique was the same between the NS and heparin groups. Furthermore, arterial cannulas could be an appropriate response to the flush test. In the heparin group, heparin (1250 units, SHP NO. 1 Biochemical and Pharmaceutical Co., Ltd., Shanghai, China) was added to 500 ml of normal saline (Baxter AG, Vienna, Austria). Finally, the heparin concentration was 2.5 U/ml in the heparin group. In the NS group, 500 ml of normal saline was used. In our hospital, blood samples were obtained from arterial cannulas for blood gas analysis. Therefore, flushing was routinely implemented after collecting blood samples. Additionally, flushing of the cannula, mobilization or both were performed if cannulas were potentially occluded.

After six months of ICU training, bedside nurses undertook the assessments of the arterial lines. To assess the usefulness of arterial cannulas, three parameters were used. (i) Patency: The cannula was sufficiently patent when the blood was back-drawn using a syringe. Otherwise, flushing or movement of the cannula was used and duly noted. (ii) Reliability of arterial pressure (AP): AP using the intra-arterial pressure measurement, compared with brachial artery cuff pressure measurement, is regarded as appropriate when the values have differences of less than ±10 mmHg between the mean AP obtained via the cannula vs the cuff [14]. (iii) Acceptable waveform: An acceptable waveform was achieved if there was an appropriate curve response to the fast flush test, indicating that, with the flush in the arterial cannula, the arterial wave disappeared, and a direct line was visible on the monitors (BeneView T9, Mindray Bio-Medical Electronics Co., Ltd., Shenzhen, China). Signs of cannulas also had to show a rapid decline to below the baseline (at approximately a 90° angle). All of the above three criteria had to be met before an arterial cannula could be considered fully functional. The arterial cannula was considered to have lost functionality if it failed to comply with any one of the above criteria. The functionality of arterial cannulas was evaluated every 6 h and at removal. To calculate the predicted risk of cardiac surgery, the European System for Cardiac Operative Risk Evaluation (EuroSCORE) was used [15]. Chest tubes were placed in the pleural cavity, pericardial cavity and mediastinum. Therefore, drainage with chest tubes was defined as drainage of the pleural cavity, pericardial cavity and mediastinum.

### Sample size and statistical analysis

Hoste EA et al. reported that the incidence of “heparin-induced coagulation disorders” was 23% [16]. The following assumptions were made: values were equivalent if the difference between proportions was no greater than 10%, alpha was 0.01, and the statistical power was 90%. Further, assuming that there was no loss of follow-up of “heparin-induced coagulation disorders” in the study, 110 patients per group were needed. After matching, the actual sample size of 296 enabled us to have sufficient power.

IBM SPSS statistical software was used (Statistics for Windows, version 22, IBM Corporation, Armonk, NY, USA) for analysis. Continuous variables were portrayed as the mean ± SD or, if appropriate, as the median with interquartile ranges (IQR). Discrete variables were depicted as frequencies (n, %). Normally continuous variables were evaluated using Student’s *t-*test, or the Mann-Whitney U nonparametric method was used for non-normally continuous variables. Continuous variables were determined to be normal in distribution by the Shapiro-Wilk test. Categorical data were equated using the chi-square test or Fisher’s exact test. The two groups were analysed using repeated measures analysis of variance (ANOVA). Differences between two groups were determined by repeat measures ANOVA with subsequent Bonferroni correction, with *P* < 0.05/n considered significant.

Some bias could exist in our study. The conclusions would be not scientific and reasonable if these biases were not eliminated. Adjustment for indication bias was further assessed using a propensity score. With the help of this method, a comparison between patients receiving HS arterial cannulas and those who received NS arterial cannulas with similar risk profiles was made possible (variables were collected when *P* ≤ 0.1 in univariate analysis, as presented in Table 1). To strengthen the reporting of observational studies, epidemiological guidelines were followed in our study [17]. For each patient, we derived the probability of NS and compared it with the patients having received HS in a 1:1 ratio and matched this ratio with the closest propensity score at up to a ± 0.01 difference. A *P* value < 0.05 was regarded as significant.

**Table 1:** Baseline and Characteristics

Variable	NS group (*n* = 170)	Heparin group (*n* = 2930)	*P* value
Age (year)	60.3 ± 9.9	58.1 ± 12.0	< 0.05
Gender (male)	114, 3.9%	958, 32.7%	0.9
Weight (kg)	66.4 ± 15.2	65.2 ± 14.1	0.3
NYHA class			0.3
I (n,%)	0	0	
II (n,%)	20, 11.8%	353, 12.0%	
III (n,%)	48, 28.2%	952, 32.5%	
IV (n,%)	102, 60.0%	1625, 55.5%	
EuroSCORE	3.3 ± 2.6	4.5 ± 3.4	< 0.001
Previous Medical History			
Acute Myocardial infarction (n,%)	103, 60.6%	1380,47.1%	0.003
Arrhythmia (n,%)	75, 44.1%	1259, 43.0%	0.8
Diabetes Mellitus (n,%)	70, 41.2%	1074, 36.7%	0.2
Chronic Renal Failure (n,%)	47, 27.6%	594, 20.3%	< 0.05
Hypertension (n,%)	123, 72.3%	1840, 62.8%	< 0.05
Liver Disease (n,%)	7, 4.1%	179, 6.1%	0.3
Smoking	91, 53.5%	1480, 50.5%	0.4
Alcohol drinking (n,%)	95, 55.9%	1834, 62.6%	0.1
Preoperative LVEF (%)	46.1 ± 4.0	45.9 ± 5.6	0.8
Preoperative LVDd (mm)	58.1 ± 9.9	59.1 ± 9.7	0.2
CPB time (minutes)	155.3 ± 66.9	132.4 ± 68.9	< 0.001
ACC time (minutes)	122.6 ± 55.0	118.8 ± 67.5	0.4
Surgical Procedures			0.1
On-Pump CABG (n,%)	29, 17.0%	373, 12.7%	
Off-Pump CABG (n,%)	46, 27.1%	634, 21.6%	
Valves (n,%)	61, 35.9%	1363, 46.6%	
CABG+ Valves (n,%)	34, 20.0%	560, 19.1%	

*NYHA*: New York Heart Association, *CPB*: Cardiopulmonary Bypass, Median (Interquartile Range), *CABG*: Coronary Artery Bypass Grafting, *ACC*: Aortic Cross Clamp, *LVDd*: Left Ventricular End-Diastolic diameter, *LVEF*: Left Ventricular Ejection Fraction, Mean ± SD, *NS*: Normal Saline

## Results

A total of 3100 patients were enrolled in this study. Amongst them, 170 patients received NS arterial cannulas, while 2930 patients received HS arterial cannulas. Therefore, the NS group had 170 patients, and the 2930 patients were included in heparin group. The baseline and demographic variables were quite different between the NS group and the heparin group (Table 1). Adjustment for selection bias was further performed using a propensity score. After propensity matching, there were 296 patients (148 in the NS group:148 in the heparin group) in the study cohort. There was no significant difference between propensity-matched groups with regard to baseline characteristics (Table 2).

**Table 2:** Baseline and Characteristics

Variable	NS group (*n* = 148)	Heparin group (*n* = 148)	*P* value
Age (year)	60.3 ± 10.0	59.5 ± 12.6	0.5
Gender (male)	104, 70.3%	115, 77.7%	0.1
Weight (kg)	65.9 ± 14.5	66.8 ± 12.6	0.6
NYHA class			0.6
I (n,%)	0	0	
II (n,%)	19, 12.8%	22, 14.9%	
III (n,%)	43, 29.0%	44, 29.0%	
IV (n,%)	87, 58.2%	83, 56.1%	
EuroSCORE	3.5 ± 2.7	3.3 ± 3.0	0.5
Previous Medical History			
Acute Myocardial infarction (n,%)	86, 58.1%	82, 55.4%	0.6
Arrhythmia (n,%)	62, 41.9%	72, 48.6%	0.2
Diabetes Mellitus (n,%)	64, 43.2%	54, 36.5%	0.2
Chronic Renal Failure (n,%)	40, 27.0%	45, 30.4%	0.5
Hypertension (n,%)	107, 72.3%	106, 71.6%	0.9
Liver Disease (n,%)	7, 4.7%	4, 2.7%	0.4
Smoking	82, 55.4%	73, 49.3%	0.297
Alcohol drinking (n,%)	90, 60.8%	85, 57.4%	0.556
Preoperative LVEF (%)	46.0 ± 4.1	46.0 ± 5.9	0.9
Preoperative LVDd (mm)	58.4 ± 9.6	58.9 ± 10.8	0.7
CPB time (minutes)	151.2 ± 65.8	148.4 ± 69.6	0.7
ACC time (minutes)	119.5 ± 54.0	115.7 ± 56.5	0.6
Surgical Procedures			0.5
On-Pump CABG (n,%)	24, 16.2%	20, 13.5%	
Off-Pump CABG (n,%)	39, 26.3%	30, 20.2%	
Valves (n,%)	53, 35.2%	72, 48.1%	
CABG+ Valves (n,%)	33, 22.3%	27, 18.2%	

*NYHA*: New York Heart Association, *CPB*: Cardiopulmonary Bypass, Median (Interquartile Range), *CABG*: Coronary Artery Bypass Grafting, *ACC*: Aortic Cross Clamp, *LVDd*: Left Ventricular End-Diastolic diameter, *LVEF*: Left Ventricular Ejection Fraction, Mean ± SD, *NS*: Normal Saline

**Fig. 1:**
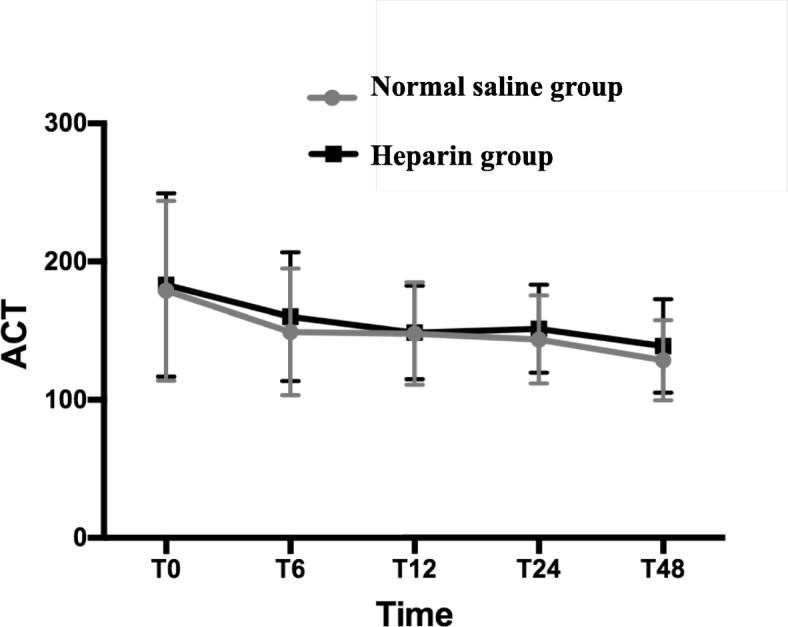
The heparin group had higher levels of ACT during the period from T0 to T48, compared with the NS group (*P* < 0.05). T0: At the end of surgery, T6: At the 6th hour after surgery, T12: At the 12th hour after surgery, T24: At the 24th hour after surgery, T48: At the 48th hour after surgery

**Fig. 2:**
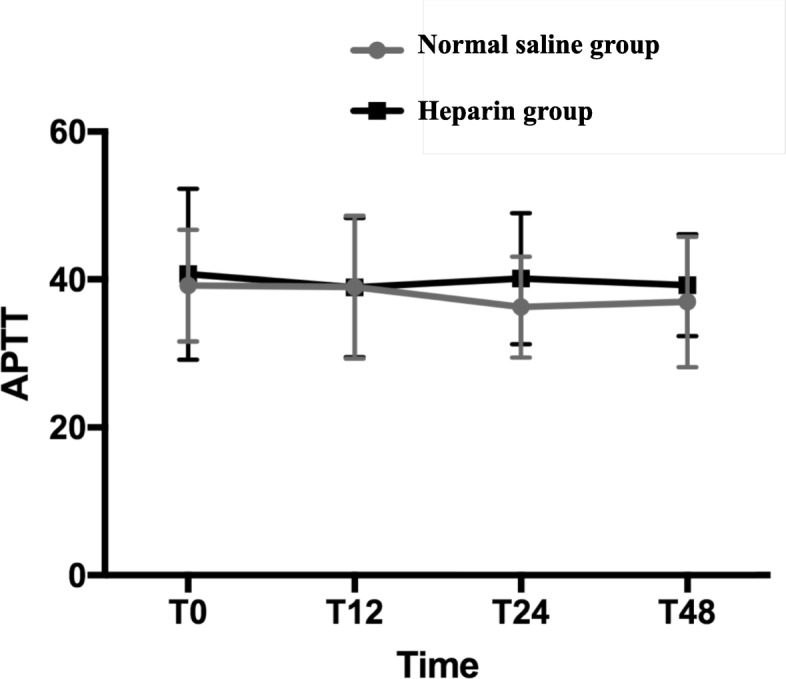
The heparin group had higher levels of APTT during the period from T0 to T48, compared with the NS group (*P* = 0.01). T0: At the end of surgery, T6: At the 6th hour after surgery, T12: At the 12th hour after surgery, T24: At the 24th hour after surgery, T48: At the 48th hour after surgery

In propensity-matched patients, the duration of arterial cannulas (58.3 ± 31.4 h vs 62.1 ± 41.4 h, *P* = 0.4) and arterial cannula occlusion (3.4% vs 2.0%, *P* = 0.5) showed no differences between the NS and heparin groups. Furthermore, flushing as well as cannulae size were not different between the NS and heparin groups. The detailed variables associated with the main variable in propensity-matched patients are presented in Table 3. As seen in Table 4, the heparin group had longer activated clotting time (Fig. 1, *P* < 0.05) and higher APTT (Fig. 2, *P* = 0.01) during the period from T0 to T48, compared with the NS group. After Bonferroni correction, heparin group had higher APTT during the period from T24 to T48 (P < 0.05/4), and had higher ACT at T48 (P < 0.05/5) compared with NS group. The heparin group had more allogeneic RBC utilization (0.1 ± 0.4 U vs 0.4 ± 1.1 U, P < 0.05) and more drainage to chest tubes (10.6 ± 9.4 ml/kg vs 13.0 ± 7.2 ml/kg, P < 0.05). In addition, more patients used RBC (3.4% vs 10.8%, P < 0.05) than in the NS group.

**Table 3:** Main variables in Propensity-matched Patients

Variable	NS group (*n* = 148)	Heparin group (*n* = 148)	*P* value
Duration of AC (hours)	58.3 ± 31.4	62.1 ± 41.4	0.4
Manipulations			
Mobilization (n, %)	12, 8.1%	7, 4.7%	0.2
Flushing(n, %)	148, 100%	148, 100%	–
Number of flushing (times)	33.7 ± 8.8	32.1 ± 7.8	0.1
Both manipulation (times)	8, 5.4%	4, 2.7%	0.2
Reasons for removal			
AC occlusion (n, %)	5, 3.4%	3, 2.0%	0.5
Discharge from ICU (n, %)	148, 100%	145, 98.0%	0.1
Patient’s death (n, %)	0	1, 0.7%	0.3
HIT (n, %)	0	3, 2.0%	0.1
Condition of the AC at removal			
Patency (n, %)	148, 100%	146, 98.6%	0.2
Reliability of AP (n, %)	148, 100%	146, 98.6%	0.2
Acceptable waveform (n, %)	148, 100%	146, 98.6%	0.2
Cannulae size			0.2
20 G, diameter: 1.1 mm (n, %)	82, 55.4%	71, 48.0%	
22 G, diameter: 0.9 mm (n, %)	44, 29.7%	49, 33.1%	
24 G, diameter: 0.7 mm (n, %)	22, 14.9%	28, 18.9%	
ICU stay time (hours)	60.3 ± 37.8	62.2 ± 43.1	0.6
Deep venous thrombosis (n, %)	9, 6.1%	5, 3.4%	0.3

Median (Interquartile Range), *NS*: Normal Saline, *ICU*: Intensive Care Unit, *AP*: Arterial Pressure, Mean ± SD, *AC*: Arterial catheter, *HIT*: Heparin-Induced Thrombocytopenia

**Table 4:** Impact of heparin on coagulation in Propensity-matched Patients

Variable	NS group (*n* = 148)	Heparin group (*n* = 148)	*P* value
ACT (second) ^**^			< 0.05
At the end of surgery (T0)	178.9 ± 65.1	183.2 ± 66.4	0.6
At the 6th hour after surgery (T6)	149.1 ± 45.7	160.0 ± 46.6	0.04
At the 12th hour after surgery (T12)	146.9 ± 37.0	148.7 ± 33.8	0.8
At the 24th hour after surgery (T24)	143.7 ± 31.8	151.4 ± 31.8	0.03
At the 48th hour after surgery (T48)	128.6 ± 29.1	138.9 ± 33.7	< 0.01
APTT (second) ^**^			0.01
T0	39.2 ± 7.5	40.7 ± 11.5	0.2
T12	39.0 ± 9.6	38.9 ± 9.4	0.9
T24	36.2 ± 6.8	40.1 ± 8.9	< 0.01
T48	37.0 ± 8.8	39.2 ± 6.9	< 0.01
Allogeneic RBC utilization (U)	0.1 ± 0.4	0.4 ± 1.1	< 0.05
Number of patients (n, %)	5, 3.4%	16, 10.8%	< 0.05
FFP utilization (ml)	29.2 ± 111.4	26.4 ± 103.4	0.8
Number of patients (n, %)	13, 8.8%	12, 8.1%	0.9
Platelets utilization (U)	0.5 ± 0.3	0.1 ± 0.4	0.5
Number of patients (n, %)	3, 2.0%	5, 3.4%	0.5
Other blood products (n, %)	2, 1.3%	6, 4.0%	0.1
Re-operation for bleeding (n, %)	0	1, 0.68%	–
Drainage of chest tube^*^ from T0 to T48 (ml/kg)	10.6 ± 9.4	13.0 ± 7.2	< 0.05
Number of LMWH used (n, %)	9, 6.1%	5, 3.4%	0.3
Protamine utilization from T0 to T24 (mg)	18.0 ± 11.2	21.1 ± 14.3	< 0.05

Mean ± SD, *RBC*: Red Blood Cell, *NS*: Normal Saline, *PT*: Prothrombin time, *APTT*: Activated Partial Thromboplastin Time, *ACT*: Activated Clotting Time, *FFP*: Fresh Frozen Plasma, *LMWH*: Low Molecular Weight Heparin, *: chest tube is placed in pleural cavity, pericardial cavity and mediastinal, **: P value was adjusted by Bonferroni correction

## Discussion

Heparinized solution is known as a common prescription for maintaining cannula patency [4, 18]. However, unnecessary exposure to heparin should be avoided because its use, even in small doses, can result in a number of complications [19, 20]. Some previous studies have indicated that cannula occlusion was not different between NS arterial cannulas and HS arterial cannulas [13, 21, 22]. Our study re-confirmed the conclusion that a marginally significant association was observed between using NS vs HS and the incidence of cannula occlusion. Moreover, acceptable arterial waveforms and the reliability of arterial pressure in NS group and heparin group were not different; the use of HS was associated with an increased risk of bleeding in patients after cardiac surgery.

Compared with the general ICU, the CICU has quite different patients. Patients who undergo cardiac surgery often receive systemic heparinization. In our hospital, intraoperatively maintaining more than 480 s of active clotting time (ACT) as a benchmark, heparin (200–400 U/kg) was used to achieve anticoagulation. At the end of CPB, protamine was initially administered to reverse heparin at a 1:1 ratio, so the activated clotting time was returned to preoperative values. Additional doses of protamine might be required if the ACT is not appropriate. In theory, we should remove previously applied heparin before applying new heparin to prevent the accumulation of medication that exceeds the prescribed dose. However, HS was routinely used to prevent arterial cannula occlusion. It can lead to unnecessary heparin exposure. Heparin, even at a low dose, can cause thrombocytopenia and bleeding [18, 23]. Upon granular review of our results, one can clearly determine that the ACT and APTT levels in both groups were significantly different but with no clinical significance since they were mainly within the normal ranges. Additionally, the amount of blood products administered in both groups was minimal. Hence, the differences might be not significant clinically. However, we routinely administered protamine to antagonize heparin if the ACT was more than 160 s, and/or drainage from the chest tube was more than 2 ml/kg/h for 1–2 h. The ACT was measured every hour on the first postoperative day. After re-analysis, the patients in the heparin group used more protamine than the NS group (Table 4). Therefore, the ACT and APTT levels in both groups were mainly within the normal ranges. In our hospital, we minimized blood product transfusions due to the high risk of acute kidney injury and transfusion-related acute lung injury in patients receiving blood transfusions. Therefore, the amount of blood products administered in both groups was minimal. Additionally, after Bonferroni correction, heparin group had higher APTT during the period from T24 to T48, and had higher ACT at T48 compared with NS group. Therefore, compared with ACT, APTT had increased more rapidly when heparin was added to normal saline solutions for maintaining the patency of arterial pressure measurement. It may be because ACT is less sensitive to residual heparin anticoagulation than APTT [24]. Finally, the patients in the heparin group had more drainage from chest tubes, higher APTT, and more protamine utilization than the NS group. In other word, the interrupted protamine may not completely eliminate the adverse effects of unmonitored heparin flushes. Hence, the differences were significant both clinically and statistically.

Del [21] et al. did not observe significant differences in arterial cannula occlusion between the two groups. They reported that the rate of arterial cannula flushing was 13–24%. However, our study found that all patients needed flushing, and the mean number of flushes was 32.9 (standard deviation: 8.3). In our hospital, blood samples were obtained from arterial cannulas for blood gas analysis. Flushing was routinely implemented after collecting arterial blood samples. In other words, we had increased flushed frequency. Heparin use, as can occur with frequent unmonitored flushes, can exert significant anticoagulant effects, which can increase the risk of bleeding, as well as alter and confound coagulation tests [16, 25, 26]. Therefore, the use of NS would result in fewer side effects from heparin-related complications, although frequent flushes were postoperatively implemented in patients undergoing cardiac surgery.

Some drugs can affect coagulation function, and they were used for prophylaxis of deep venous thrombosis. In our centre, low molecular weight heparin (LMWH) was used to prevent deep venous thrombosis, which should be monitored for daily by ultrasonography to assure that the LMWH is successfully administered early. Moreover, early rehabilitation intervention was usually implemented as soon as possible. Further, we would encourage patients to walk if they had been weaned from mechanical ventilation. LMWH was only prophylactically used to prevent deep venous thrombosis in long-term bedridden patients. Table 3 shows that the ICU length of stay was not different between the groups. There might be a few long-term bedridden patients in our study population. Tables 3 and 4 indicated that deep venous thrombosis and the amount of LMWH used were not different between the NS and heparin groups. Therefore, LMWH utilization might not have influenced the outcomes.

A systematic review [22] reported that HS could be chosen to prevent occlusion in patients with short lengths of ICU stay (< 30 days). However, a randomized, controlled trial suggested that NS could be used to maintain patency of arterial cannulas [13] for the short term (3 days) after cardiac surgery. In our study, the mean length of ICU stay was 61.3 h (standard deviation: 40.5), indicating that the turnover rates of CICU beds might be higher than in the general ICU. Therefore, a short length of CICU stay could result in unnecessarily using HS arterial cannulas to prevent arterial cannula occlusion. In general, the use of heparin should be controlled in patients after cardiac surgery.

## Study limitations

Our study design involves one centre’s experiences with the inherent disadvantages of an observational study, which is highly prone to bias. This observational study could be influenced by potential biases. We used propensity score matching to avoid these biases. However, factors that affect assignment to treatment and outcomes but that cannot be observed cannot be accounted for in the matching procedure. Any hidden bias due to latent variables might remain after matching, which could lead to some statistical faults. Furthermore, with this analysis, we remove a large amount of patients from the analysis, but may have elevated statistical errors. The multiple statistical analyses was also undertaken in our study. The outstanding criticism is the inflated risk of Type I error because of multiple statistical analyses. It may have adverse effects on our study. Additionally, the reliability of the pressure measurement was tested against the brachial artery pressure measure by cuff and sphygmomanometer, which is questionable in the setting of cardiac surgery because patients often experience vasodilation as a result of systemic inflammatory response syndrome, and there could be a large difference in arterial blood pressure measured at the radial artery compared with more centrally, such as the brachial artery. The cuff arterial pressure measured on the arm is dependent on the ratio of the cuff size to arm size. These limitations of this assessment are important and should be acknowledges.

## Conclusion

Based on the results of our study, the addition of heparin to normal saline for flushing arterial pressure monitoring cannulae did not reduce the incidence of catheter thrombosis and result in a very small but statically significant in increase in ACT and APPT.

## Abbreviations

ACT: Activated clotting time
APTT: Activated partial thromboplastin time
CPB: Cardiopulmonary bypass
HICD: Heparin-induced coagulation disorders
HS: Heparinized saline
NS: Normal saline
